# Cochlear Dummy Electrodes for Insertion Training and Research Purposes: Fabrication, Mechanical Characterization, and Experimental Validation

**DOI:** 10.1155/2015/574209

**Published:** 2015-07-05

**Authors:** Jan-Philipp Kobler, Anandhan Dhanasingh, Raphael Kiran, Claude Jolly, Tobias Ortmaier

**Affiliations:** ^1^Institute of Mechatronic Systems, Gottfried Wilhelm Leibniz Universität Hannover, Appelstraße 11a, 30167 Hanover, Germany; ^2^MED-EL Medical Electronics, Fuerstenweg 77a, 6020 Innsbruck, Austria

## Abstract

To develop skills sufficient for hearing preservation cochlear implant surgery, surgeons need to perform several electrode insertion trials in *ex vivo* temporal bones, thereby consuming relatively expensive electrode carriers. The objectives of this study were to evaluate the insertion characteristics of cochlear electrodes in a plastic scala tympani model and to fabricate radio opaque polymer filament dummy electrodes of equivalent mechanical properties. In addition, this study should aid the design and development of new cochlear electrodes. Automated insertion force measurement is a new technique to reproducibly analyze and evaluate the insertion dynamics and mechanical characteristics of an electrode. Mechanical properties of MED-EL's FLEX^28^, FLEX^24^, and FLEX^20^ electrodes were assessed with the help of an automated insertion tool. Statistical analysis of the overall mechanical behavior of the electrodes and factors influencing the insertion force are discussed. Radio opaque dummy electrodes of comparable characteristics were fabricated based on insertion force measurements. The platinum-iridium wires were replaced by polymer filament to provide sufficient stiffness to the electrodes and to eradicate the metallic artifacts in X-ray and computed tomography (CT) images. These low-cost dummy electrodes are cheap alternatives for surgical training and for *in vitro, ex vivo*, and *in vivo* research purposes.

## 1. Introduction

Cochlear implants (CI) are currently the only solution to restore hearing in patients with profound deafness. Electrodes, which are one of the important components of CI, have more than one stimulating channel placed inside the scala tympani to elicit action potentials in the auditory neural tissues tonotopically. Recently, the indication for CI has been extended to partial deafness [1, 2]. As a result, hearing preservation (HP), by soft surgical techniques to protect the intracochlear fine structures, is the latest trend especially for patients with good residual hearing in the mid- to low-frequency apical region. Hearing preservation may also be important for future therapies that may need these fine structures in place for the regeneration of the neural fibers [3]. Although the correlation between the intracochlear trauma due to insertion and the conservation of residual acoustic hearing has not been distinctly stated, it is assumed that atraumatic electrode insertion is essential to prevent neuronal cell death and trauma to the internal structures [4–7]. One of the main factors influencing the outcome of soft surgical procedures is how surgeons handle and push the electrode gently inside the scala tympani [8–10]. Performing this soft surgical technique requires a high level of surgical skill and a wealth of hands-on experience.

Cochlear implant electrodes are made of biocompatible conducting wires and electrode contacts housed within a flexible biocompatible elastomer. The electrode contacts (typically made of platinum/iridium or gold) are electrically connected to the wires for operationally contacting intracochlear structures of the CI user to deliver the electrical signal. The soft elastomers generally preserve the basilar membrane and the organ of Corti during the insertion process. Electrode atraumaticity depends on the stiffness of the wires and the smoothness of the elastomer. The size, shape, stiffness, and length of the electrodes vary among manufacturers [11].

Surgeons need to perform several electrode insertion trials in* ex vivo* temporal bones to develop the skills necessary for successful HP surgery in a patient. The electrodes required for such training, however, are expensive due to the high cost of the raw materials and the manufacturing.

The metallic artifact that cochlear electrodes create with clinical and *μ*CT imaging is also a problem [12–14]. For many* ex vivo* research purposes and medical studies, for example, studying the impact of electrode insertion on the intracochlear fine structures or cochlear duct length measurement, the metallic artifact is a great hindrance. Such temporal bone studies also help to evaluate the mechanical properties of newly developed electrode arrays. This includes evaluating the damage to the basilar membrane, lateral, and medial cochlear walls due to the insertion of an electrode array, the positioning of the electrode array within the scala, and so forth. In radiographs, however, metallic artifacts from the electrode array overshadow the fine tissue structures.

In order to address the abovementioned problems, we developed silicone dummy cochlear electrodes of various array lengths. If successful, cost-effective, easy-to-fabricate dummy electrodes could be used for* ex vivo* research purposes and for insertion training.

The objective of this study was to develop electrode arrays with comparable mechanical properties to those of the FLEX series (MED-EL GmbH, Innsbruck, Austria) by using radio opaque polymer filament to replace the metallic wires. The mechanical properties of these dummy electrodes were determined using an automated insertion tool with integrated force-sensing capability [15]. This tool was originally developed for minimally invasive cochlear implant surgery [16–18], but it also serves as an instrument for reproducible electrode characterization within a bench top setup since it minimizes variations in the insertion procedure due to human interaction [19]. The measured insertion force, combined with video documentation of the automated insertions, was considered to compare characteristics of the dummy electrodes to those of their commercially available wire-based counterparts.

## 2. Materials and Methods

### 2.1. Materials

To fabricate the dummy electrodes, we used two polymer filaments that varied in material and thickness, obtained from Goodfellow Cambridge Ltd., Huntingdon, United Kingdom. Monofilament made of polyethylene terephthalate (PET) had a thickness of 100 *μ*m and the monofilament made of fluorinated ethylene propylene copolymer (FEP) had a thickness of 280 *μ*m. Medical grade silicone elastomer from NuSil Technology LLC, Carpinteria, CA, USA, was used as electrode carrier and 10% iridium blended platinum wire (×1) with a thickness of 25 *μ*m was used as the electrode tracker, which helps to locate the dummy implant in CT images.

### 2.2. Electrode Fabrication

We fabricated the dummy electrode array in 28 mm, 24 mm, and 20 mm lengths, equivalent to the FLEX^28^, FLEX^24^, and FLEX^20^ electrodes, commercially available brands from MED-EL GmbH. The inner surface of the mold was first painted with a thin layer of silicone followed by the polymer filament placement together with a platinum wire (10% iridium). The mold halves were closed, injected with silicone, and cured at 110°C for 4 hours. The mold halves were separated to remove the cured electrode.  Table 1 gives an overview of the manufactured dummy electrodes for consideration in this study.

**Figure 1:**
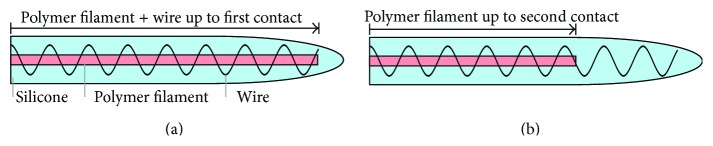
Cross-section of dummy electrodes: standard fabrication procedure (a) and fabrication using filament with reduced length (b).

**Figure 2:**
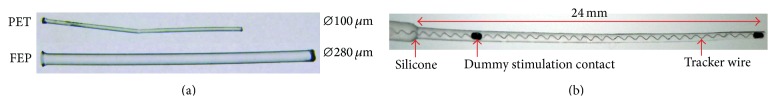
Samples of PET and FEP filament (a), fabricated dummy electrode F24 01 (b). The stimulation contacts (not connected) and the tracker wire are included to facilitate localization of the electrode in CT images. The opaque PET filament is not visible in this picture.

With the standard fabrication procedure, the wire and the polymer filament were inserted up to the position of the first stimulation contact. This contact is not functional in the dummy electrodes but helps to localize the tip of the implant in X-ray and CT images. To analyze the effects of a more flexible electrode tip, one prototype (F28 03) was manufactured using filament that went up to the original position of the second stimulation contact (see also Figure 1).  Figure 2 shows one such dummy electrode (F24 01) which was fabricated according to the dimensions of the FLEX^24^ model.

In order to assess the manufacturing costs of the dummy electrodes, the polymer filament, wires, and silicone required for fabrication are considered. Based on the current pricing of these components, the manufacturing costs of the dummy electrodes are estimated to be approximately 2% of those required to fabricate the commercially available models.

### 2.3. Insertion Force Measurement

In cochlear implantation surgery that aims for HP, trauma associated with electrode insertion needs to be minimized to preserve both the delicate intracochlear membranes and the sensory hair cells. Arguably, insertion trauma and loss of residual hearing are due to intracochlear forces applied during electrode deployment. It is widely accepted that the magnitude of such insertion forces correlates with the amount of intracochlear trauma [20, 21]. Considering straight electrodes, insertion forces depend on the mechanical properties of the electrode carriers and other parameters which are not assessed but kept constant in this study [22, 23]. Here, the measured insertion force is considered to compare the dummy electrodes to their commercial counterparts.

**Figure 3:**
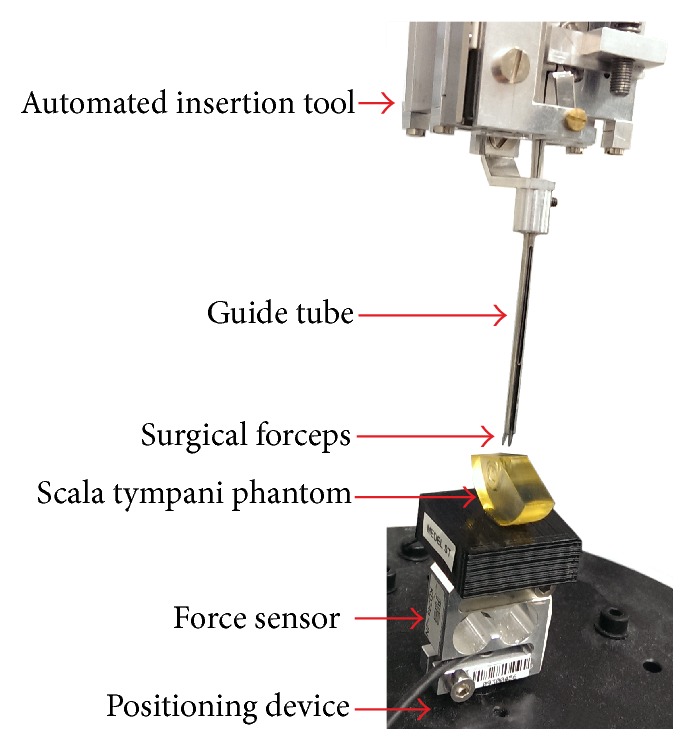
Overview of the experimental setup for automated insertion studies and components involved.

Insertion force measurements, for which an automated insertion tool was used, were performed according to the experimental setup proposed by Kobler et al. [15]. The components involved are given in Figure 3. The automated insertion tool was originally designed for both straight and preformed electrode carriers, which are preoperatively straightened by a platinum wire. The tool therefore incorporates two linear actuators to independently actuate the implant feed and the position of the straightening wire. Since only straight electrodes were considered in this study, one actuator remained passive during the trials. The actuators based on piezo technology provide a traveling range of 45 mm and a position accuracy of 1 *μ*m (SL1560, SmarAct GmbH, Oldenburg, Germany). The electrode carrier to be inserted can be grasped by surgical forceps with flat jaws attached to the implant actuator. This grasping mechanism is covered by a u-shaped guide tube to provide guidance of the implant during the insertion process.

All insertions were performed into an acrylic scala tympani phantom developed by MED-EL GmbH and based on histological human temporal bone data. Further specifications of the phantom are given in Leon et al., 2014 [24]. To measure insertion force, we used a commercially available, s-shaped, single axis load cell with a measuring range of up to 2 N (KD24S-2 N), onto which the phantom was placed. This sensor was also mounted on a passive positioning device, which allowed the precise adjustment of the phantom's position and orientation with respect to the insertion tool and, therefore, the feed motion of the electrode carrier. The load cell was operated using a carrier frequency amplifier system (MGCplus and ML55B, Hottinger Baldwin Messtechnik, Darmstadt, Germany). The analogue output of the measurement amplifier was connected to a 16-bit DAQ-System (NI USB-6251 BNC, National Instruments, Austin, Texas, USA) and sampled at a rate of 1,000 Hz. The resulting resolution of the measuring system was well below 1 mN.

### 2.4. Insertion Protocol

**Figure 4:**
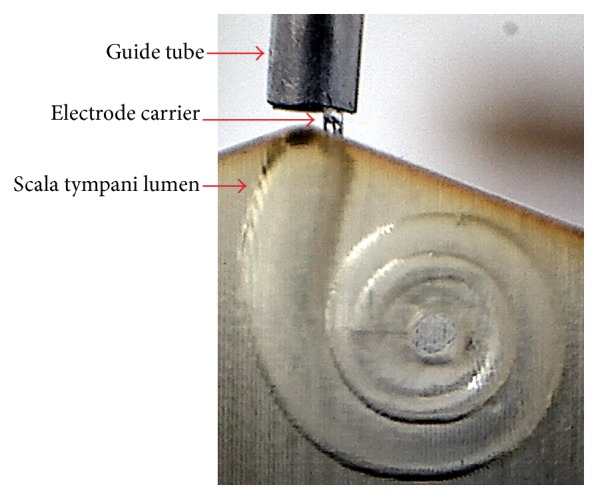
Initial position and orientation of the implant with respect to the phantom.

To derive a gold standard for the evaluation of the dummy electrodes, the insertion force profiles of the commercially available FLEX^28^, FLEX^24^, and FLEX^20^ electrodes were recorded. For each electrode, the following procedure was followed: (1) the implant was loaded into the automated insertion tool, (2) the phantom was filled with a soap solution and the electrode was positioned just inside the opening of the lumen (see also Figure 4), and (3) the automated insertion was performed at a constant velocity of 0.5 mm/s while insertion forces were acquired simultaneously. Each tested electrode carrier was inserted five times while maintaining both the grasping and the initial position of the implant.

Considering the commercial models, three electrodes of each type were tested according to the procedure described above, resulting in 15 insertions per model. The insertion depth, that is, the linear displacement from the electrode's initial position, was considered as 27 mm for the FLEX^28^, 23 mm for the FLEX^24^, and 19.5 mm for the FLEX^20^.

The same protocol was followed to evaluate the dummy electrodes and compare them with their commercially available counterparts. Each prototype, listed in Table 1, was inserted five times. The fourth insertion of each series was documented using a digital video microscope. It is important to note that, in order to minimize measurement errors due to human intervention, the relative alignment between the insertion tool and the phantom was maintained throughout the study.

## 3. Results and Discussion

80 automated insertions (45 insertions of commercially available models and 35 insertions of dummy electrodes) were successfully performed.

**Figure 5:**
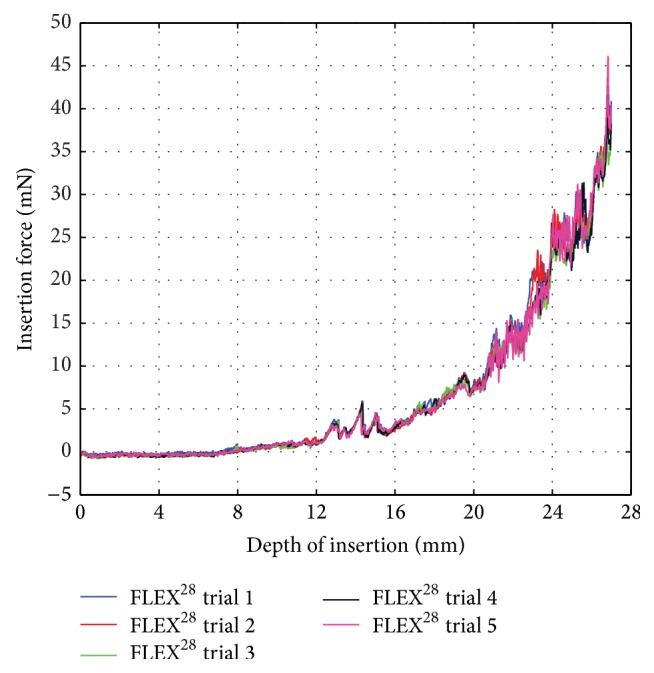
Insertion force curves measured during five consecutive insertions of the same FLEX^28^ electrode.

To assess the repeatability and reproducibility of an insertion force curve using the proposed experimental setup, Figure 5 gives the force data obtained during five consecutive measurements of the same FLEX^28^ electrode. The results of the five trials were very similar and strongly correlated. Here, the smallest correlation coefficient resulting from a pairwise comparison between the five force curves equaled 0.86. When averaging the force curves over insertion depth, the maximum standard deviation, 4 mN at a depth of 25.88 mm, is low. This confirms that sufficient repeatability was achieved.

**Figure 6:**
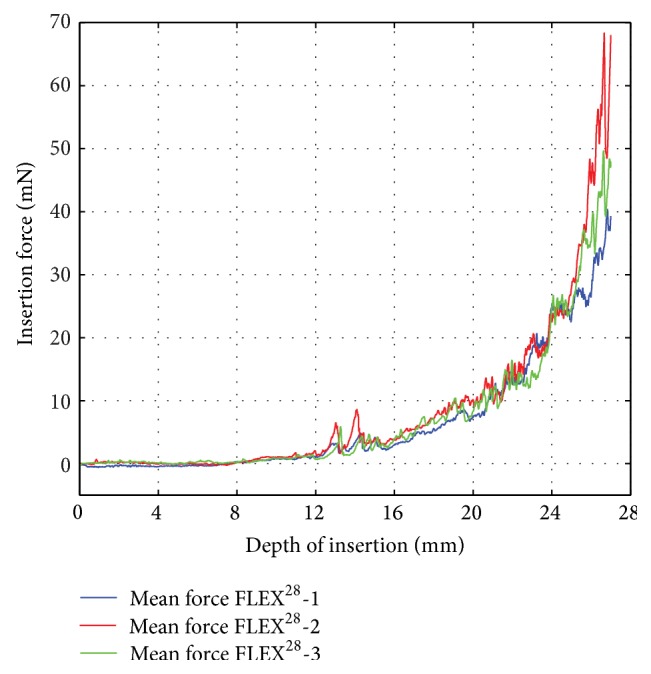
Mean insertion forces of three similar FLEX^28^ electrodes.

Due to the similarity of the results, the mean insertion force over the five trials was considered a suitable approximation for characterization of one electrode.  Figure 6 gives the mean insertion force curves of three similar FLEX^28^ electrodes (denoted as FLEX28-1, FLEX28-2, and FLEX28-3). Again, the presumably identical electrodes exhibit similar characteristics up to a depth of approximately 25 mm and then diverge slightly during the final three millimeters of the insertion. Because each electrode is manufactured by hand, such marginal variations are to be expected.

**Figure 7:**
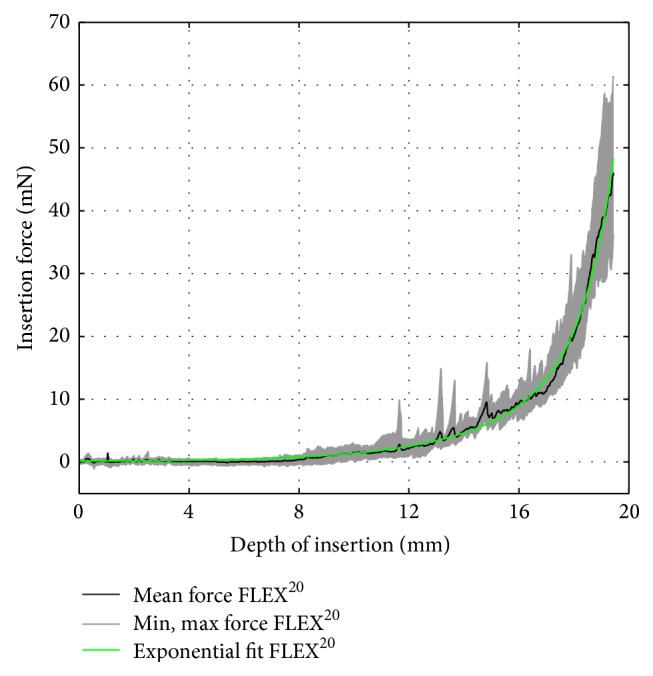
FLEX^20^ insertion force model.

**Figure 8 fig8:**
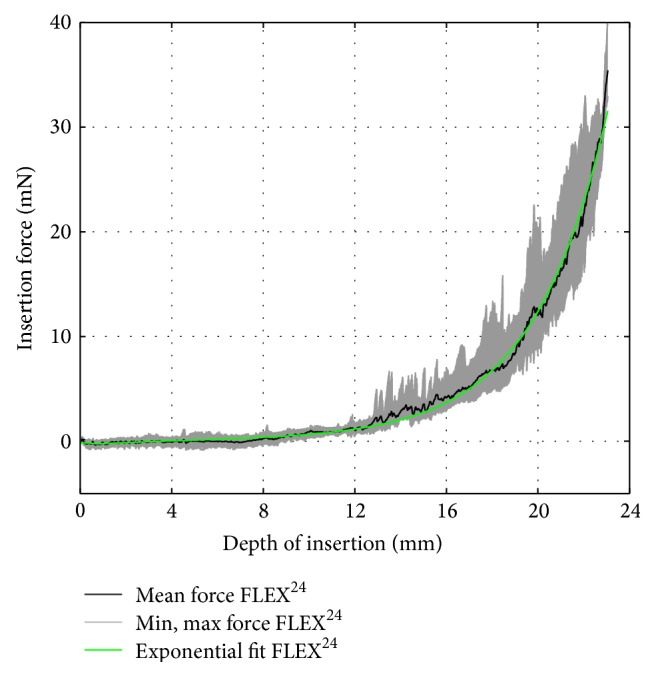
FLEX^24^ insertion force model.

**Figure 9:**
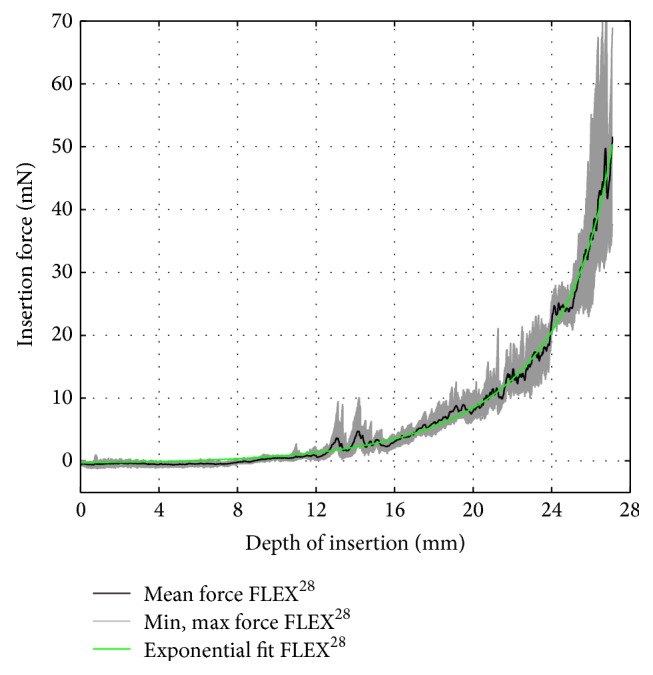
FLEX^28^ insertion force model.

To compare the commercially available electrodes to their corresponding dummy electrodes, the following vectors were computed based on the recorded data, that is, 15 insertion force curves per electrode: the arithmetic average and the minimum and maximum insertion force over insertion depth. Furthermore, the arithmetic average curve was approximated by fitting an exponential function of the following formula:

(1) $$\begin{array}{c} f_{i}\left(d_{i}\right)=ae^{bd_{i}}+ce^{dd_{i}} \end{array}$$

to obtain a compact representation of the relation between insertion force *f*_*i*_ and insertion depth *d*_*i*_. The choice of an exponential function was motivated by the following physical assumptions: given a straight, flexible electrode carrier, the measured insertion force is believed to depend on the radius of curvature of the scala tympani's outer wall, which can be modeled by a logarithmic spiral whose radius is defined as an exponential function of the spiral angle, that is, the insertion angle in this case [25]. Furthermore, according to the Capstan equation [26], the frictional forces between the outer wall of the scala tympani and the silicone carrier, which make a significant contribution to the total insertion force [10], increase exponentially depending on the insertion angle. For the commercially available electrodes, the computed data are given in Figures 7 [Fig fig8]–9. The results of the curve fitting procedure can be found in Table 2. The coefficients of determination (*r* ^2^) confirm that the measured insertion force curves of the commercially available electrodes were suitably approximated by an exponential function of the chosen form [27].

**Figure 10:**
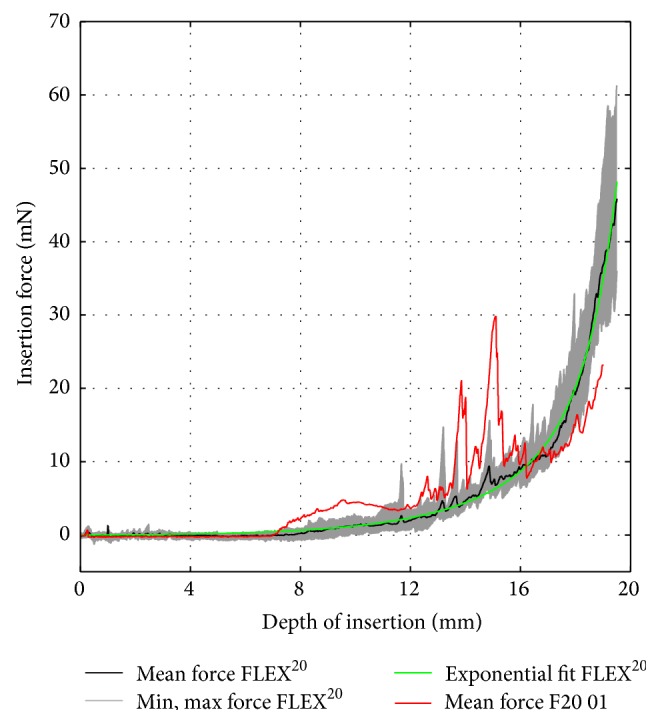
Insertion force comparison between commercially available FLEX^20^ electrode (black curve) and PET filament dummy (red curve).

**Figure 11:**
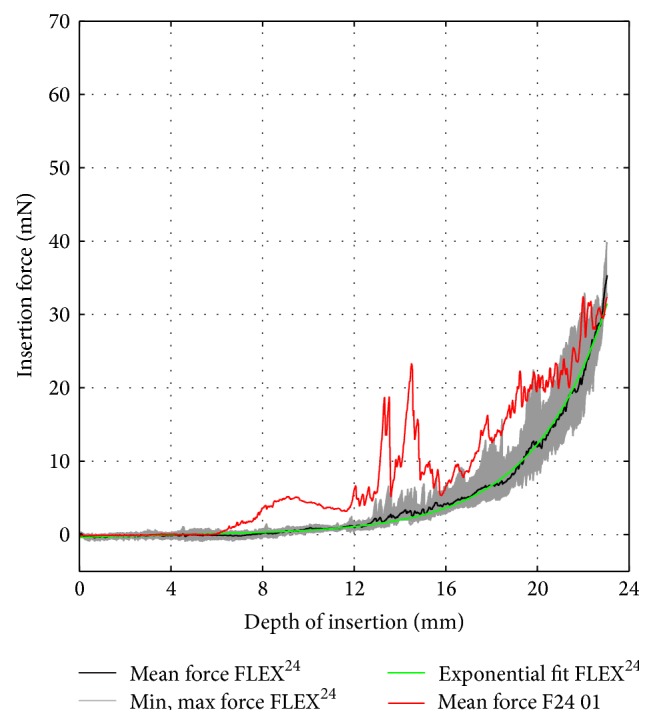
Insertion force comparison between commercially available FLEX^24^ electrode (black curve) and PET filament dummy (red curve).

**Figure 12:**
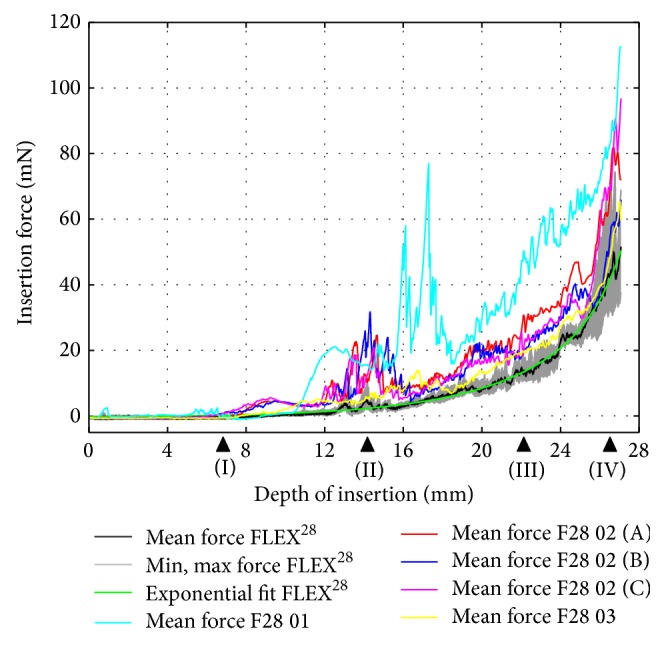
Insertion force comparison between commercially available FLEX^28^ electrode (black curve) and dummy electrodes characterized by PET filament (red, blue, and magenta curves), FEP filament (cyan curve), and PET filament with reduced length (yellow curve).

**Figure 13:**
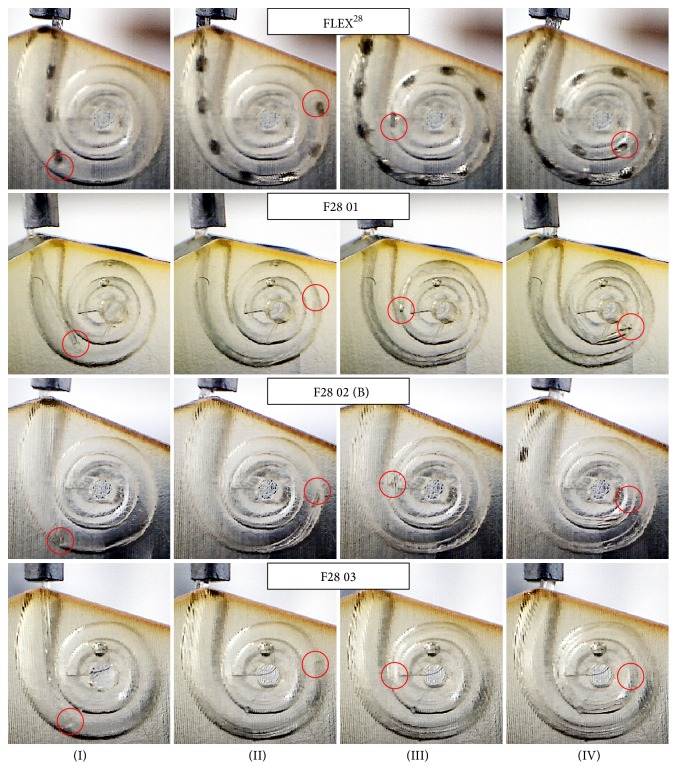
Documentation of the insertion procedure at characteristic insertion depths (see also Figure 12) for FLEX^28^ and three different dummy electrode types. Due to the opaque material of the dummy electrodes, the position of the tip is indicated by a red circle.

A similar evaluation procedure was followed for the dummy electrodes. For each prototype, the mean insertion force over insertion depth was computed by averaging the recorded data of five consecutive trials.  Figure 10 (F20 01) and Figure 11 (F24 01) compare the dummy electrodes to their commercially available counterparts. A similar comparison is given in Figure 12 for all electrodes made according to the FLEX^28^ specifications. Furthermore, the insertion procedures of FLEX^28^, F28 01, F28 02 (B), and F28 03 at characteristic insertion depths (indicated in Figure 12) are given in Figure 13. Regardless of electrode length, the insertion force curves of the dummy electrodes exhibit similar characteristics. An increase in insertion force for the dummy electrodes at a depth of approximately 6.5–8 mm was observed (see also position (I) in Figure 13), which did not occur when measuring the commercially available electrodes. This was due to the initial contact of electrode tip on the outer wall of the scala tympani phantom. The polymer filament inside the silicone carrier gives a homogenous mechanical strength from the base to the apex of the electrode, although arrays with a flexible tip are generally preferable for atraumatic insertion. Because the tip of the dummy electrode is slightly stiffer and more rigid than is ideal, the insertion force measurement showed two characteristic peaks at 14 mm and 16 mm, as, at this insertion depth, the tip of the electrode had to make an almost complete turn (position (II) in Figure 13). The measured insertion force was mainly composed of (1) the force needed to bend the electrode and (2) Capstan friction due to the silicone carrier being in contact with the inner surface of the scala tympani phantom. With the polymer filament inside the dummy electrode, the mechanical property of the dummy electrode is similar from the base to the apex of the electrode, which is not the case with the regular wire-based electrode. This could be the reason for the peaks in the insertion forces at different insertion depths. The results also reveal that, due to the characteristics mentioned above, an exponential function cannot approximate the insertion force curves of the dummy electrodes in a suitable way.

To enable a quantitative comparison between the insertion force curves of the commercially available and the dummy electrodes, the following metrics were considered and computed:

- ** M1** was defined as the root-mean-square-error (RMSE) between the mean insertion force of the commercial electrode and the mean insertion force of the dummy electrode.
- ** M2** was defined as the RMSE between the exponential fit of the commercially available electrode and the mean of the dummy electrode. This metric was expected to be similar to** M1** in case the exponential function was a good approximation of the force curves.
- The third metric was based on the assumption that the mean insertion forces of a dummy electrode should be higher than the minimum and lower than the maximum insertion forces of its commercially available counterpart. For each insertion depth the following conditions were checked:
  1. If the mean force of the dummy electrode was above the maximum of the commercially available electrode's mean force, an error value was defined as the absolute deviation from the maximum.
  2. If the mean force of the dummy electrode was below the minimum of the commercially available electrode's mean force, the error value was defined as the absolute deviation from the minimum.
  3. If the mean force of the dummy electrode was between the minimum and maximum insertion force of the commercially available electrode's mean force, the error value was equal to zero.
- Finally,** M3** was defined as the RMS of the error values described above.

All of the considered metrics penalize deviations of the dummy electrodes' insertion forces from the references of their commercially available counterpart; therefore, the lower the metric is, the closer the dummy electrode is to its commercially available counterpart.  Table 3 lists the values of these metrics for all considered dummy electrodes.

All considered metrics yielded a similar “ranking” of the dummy electrodes. According to the values, electrode F28 01, which was made using FEP filament, is the least suitable prototype in terms of insertion force. This rating is in good agreement with the results seen in Figure 12 and confirms that the measured force was considerably higher compared to FLEX^28^. The prototypes made using PET filament (F20 01, F24 01, and F28 02) generally exhibited smaller deviations from the force curves of the commercially available electrodes. The best match, however, compared to the reference was achieved using the specifications of the shortest electrode carrier, that is, FLEX^20^, while the divergences increased for FLEX^24^ and FLEX^28^. Referring to the metrics in Table 3, the force curve of prototype electrode F28 03, which is characterized by PET filament with a reduced length, was the closest to that of the commercially available FLEX^28^ implant. According to our observations, the softer tip of the silicone carrier brings the mechanical properties of this dummy electrode closer to those of the regular, wire-based model. Consequently, when the first contact with the outer wall of the phantom takes place, the soft tip kinks and deflects (see also position (I) in Figure 13). As a result, the characteristic increase in insertion force, usually observed at an insertion depth of 6.5 to 8 mm, is delayed by approximately 3 mm, since the filament is pushed against the outer wall of the phantom. This increase was, however, less distinct than that of the rest of the measured dummy electrodes.

## 4. Conclusions

To evaluate the mechanical properties of MED-EL's FLEX series electrodes, we performed the insertion force measurement in a plastic scala tympani model with dummy electrodes of equivalent mechanical characteristics. The experimental setup, comprising of an automated insertion tool, was suitable for electrode characterization and comparison since it provided sufficient reproducibility of insertion force curves and low standard deviation among consecutive trials. Reproducibility was mainly achieved by keeping constant the relevant parameters that influence insertion force, for example, the alignment of insertion tool and phantom. Furthermore, automation of the insertion procedure minimizes the required amount of human intervention between consecutive trials, which, in turn, leads to reduced variations in the experimental results.

Statistical analysis and curve fitting showed that the measured insertion force of straight, commercially available electrodes increased exponentially with the insertion depth. Due to the curve fitting procedure, a compact, formal representation of this relation is given in this paper, taking into account three commercially available electrode models. The parameters of the exponential functions are considered a valuable contribution to further studies on insertion force since the curves given in this paper can easily be reproduced by other researchers.

Further studies are required to determine the factors affecting insertion force, which include manufacturing variability, thickness of metallic wire, contact spacing, tip diameter, lubricants, and insertion speed. Based on the deviation of the quantitative comparison of dummy and reference electrodes, the F28 03 was the dummy electrode closest to the reference in terms of insertion force. The use of PET filament with reduced length (as shown in Figure 1) was therefore the most suitable creation technique. It serves as a basis for further optimization, which aims at matching the force curves of commercially available electrodes even closer with those of the dummy electrodes.

Future studies are planned to evaluate (1) the mechanical properties by nanoindenter-based bending test and (2) temporal bone insertion followed by *μ*CT imaging to assess the traumaticity and the radio opaque characteristics of the dummy electrodes. In this context, we also aim for a direct comparison between dummy electrodes and commercial models in the hands of surgeons to determine the handling characteristics and the suitability for training purposes. The insertion tool used in this study can also be used for benchmarking the traumaticity of electrodes and can serve in the design and development of new electrodes.

## Figures and Tables

**Table 1:** Characteristics of dummy electrodes fabricated in this study.

Reference	Dummy identifier	Characteristic	Number of prototypes
FLEX^28^	F28 01	FEP filament	1
	F28 02	PET filament	3
	F28 03	PET filament with reduced length	1

FLEX^24^	F24 01	PET filament	1

FLEX^20^	F20 01	PET filament	1

**Table 2:** Curve fitting parameters of MED-EL electrode insertion forces.

Name	*a*	*b*	*c*	*d*	RMSE (in mN)	*r* ^2^
FLEX^20^	0.063	0.303	7.120 · 10^−8^	1.009	0.6839	0.9942
FLEX^24^	0.025	0.310	−0.531	−0.221	0.4906	0.9952
FLEX^28^	2.056 · 10^−8^	0.754	0.157	0.202	0.8724	0.9934

**Table 3:** Considered metrics for the evaluation of dummy electrodes.

Dummy identifier	**M1** (in mN)	**M2** (in mN)	**M3** (in mN)
F20 01	4.5169	4.6307	3.1427
F24 01	5.2742	5.3238	3.5782
F28 01	21.9785	21.9664	19.1608
F28 02 (A)	10.1176	10.1219	7.1303
F28 02 (B)	6.9967	7.0418	5.2088
F28 02 (C)	8.8904	8.9263	5.5272
F28 03	4.0276	3.9461	2.3394
